# Rhodopyran, a carboxylated hexahydrocyclopenta[*b*]pyran from *Rhodococcus*

**DOI:** 10.3762/bjoc.22.89

**Published:** 2026-07-28

**Authors:** Enjuro Harunari, Sara Fushimi, Yasuhiro Igarashi

**Affiliations:** 1 Biotechnology Research Center and Department of Biotechnology, Toyama Prefectural University, 5180 Kurokawa, Imizu, Toyama 939-0398, Japan

**Keywords:** bicyclic compound, cyclopenta[*b*]pyran, natural products, *Rhodococcus*, specialized metabolites

## Abstract

A new bicyclic metabolite, rhodopyran (**1**), was isolated from the culture broth of *Rhodococcus* sp. strain RD066637. The planar structure was elucidated by 1D/2D NMR analyses and HRMS spectrometry, and the relative configuration was assigned from vicinal coupling constants and NOESY correlations, while the absolute configuration remains undetermined. Rhodopyran represents a structurally unusual carboxylated hexahydrocyclopenta[*b*]pyran and expands the known metabolite diversity of *Rhodococcus*, highlighting *Rhodococcus* as a source of uncommon natural-product scaffolds.

## Introduction

Actinomycetes have long been recognized as prolific producers of structurally diverse specialized metabolites, with *Streptomyces* historically serving as the major source of chemically characterized compounds. In contrast, non-streptomycete actinomycetes remain comparatively underexplored, and the genus *Rhodococcus* is one such promising but less extensively investigated source.

*Rhodococcus* has traditionally been investigated mainly for its broad catabolic and biotransformation capabilities. However, genome-based studies have indicated that *Rhodococcus* also possesses a substantial and largely uncharacterized capacity for specialized metabolite biosynthesis. Comparative genomic analysis has shown that many predicted *Rhodococcus* biosynthetic gene cluster families are clade-specific and lack close homology to gene clusters for known natural products [1]. More recent large-scale genome mining has further highlighted the broad unexplored biosynthetic potential of *Rhodococcus* [2].

Genome analyses of *Rhodococcus* have revealed diverse biosynthetic gene clusters, including nonribosomal peptide synthetase (NRPS), polyketide synthase (PKS), and terpene-related pathways [3–4]. Comparative genomic and taxogenomic studies have also highlighted the genomic variability and mosaic genomic architecture of *Rhodococcus* [5–6]. In addition, antibiotic biosynthesis following horizontal gene transfer from *Streptomyces* to *Rhodococcus* has been demonstrated for rhodostreptomycins [7].

**Figure 1:**
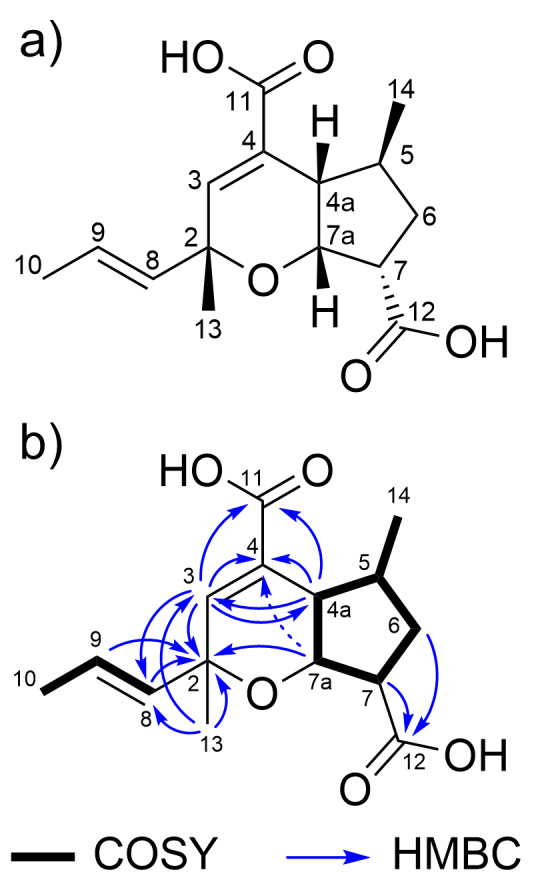
Structure and selected 2D NMR correlations of rhodopyran (**1**). (a) Structure of **1** with relative configuration; one enantiomer is shown. (b) Selected COSY correlations and key HMBC correlations. The dashed blue arrow indicates the weak but observed HMBC correlation from H-7a to C-4.

Chemically characterized *Rhodococcus* metabolites include rhodostreptomycins [7], a quinoline antibiotic [8], rhodopeptins [9], lariatins [10], rhodochelin [11], and the pluramycin-class metabolite rausuquinone [12]. These examples illustrate the chemical diversity within the genus *Rhodococcus*, but its metabolite space remains far less explored than that of *Streptomyces*. In our continuing cultivation-based survey of metabolites from *Rhodococcus*, *Rhodococcus* sp. strain RD066637 was found to produce a new cyclopenta[*b*]pyran metabolite, rhodopyran (**1**) (Figure 1). Herein, we describe the isolation, structure elucidation, relative configuration, and structural uniqueness of **1**.

## Results and Discussion

Strain RD066637 was precultured in ISP 2 medium at 30 °C and then transferred to A-3M production medium (30 °C, 5 days). The whole culture was extracted with 1-BuOH to broadly recover metabolites as part of our search for new compounds. The concentrate was fractionated by silica gel column chromatography, and preparative HPLC afforded rhodopyran (**1**, 22.7 mg from 2 L of culture, corresponding to 0.18% of the 1-BuOH extract). An uninoculated A-3M medium control was incubated for 5 days and processed under identical conditions. HPLC analysis showed no peak corresponding to rhodopyran (**1**) at the retention time of **1** in the medium control (Figure S9, Supporting Information File 1), indicating that **1** was not a pre-existing medium component detectable under the present HPLC conditions. Although several additional UV-detectable peaks were observed in the culture extract, they were either also present in the medium control, present only in trace amounts, or did not yield sufficient material for complete structure elucidation. Therefore, this study focused on compound **1**.

Compound **1** was obtained as an optically active colorless powder ([α]_D_^25^ −69 (*c* 0.01, MeOH)). The molecular formula of **1** was determined to be C_15_H_20_O_5_ based on HRESIQTOFMS data in the negative-ion mode: *m*/*z* 279.1237 [M − H]^−^ (calcd for C_15_H_19_O_5_^−^, 279.1238; Δ −0.4 ppm). The IR spectrum showed a strong band at 1688 cm^−1^, consistent with carboxyl carbonyl groups. The ^1^H NMR spectrum displayed a deshielded olefinic singlet at δ_H_ 6.72; a pair of coupled olefinic protons at δ_H_ 5.73 and 5.48; an oxygenated methine at δ_H_ 4.36; a deshielded methine at δ_H_ 3.16; methine signals at δ_H_ 2.36 and 1.97; methylene resonances at δ_H_ 2.53 and 1.44; and three methyl groups at δ_H_ 1.67, 1.27, and 1.11. The ^13^C NMR spectrum exhibited 14 signals, including two carbonyl carbons (δ_C_ 176.1, 170.6), four olefinic carbons (δ_C_ 142.9, 135.5, 130.2, 125.0), an oxygenated quaternary carbon (δ_C_ 75.6), an oxygenated methine (δ_C_ 77.2), and three methyl carbons (δ_C_ 24.3, 21.1, 17.9) (Table 1). The remaining carbon resonance (C-7, δ_C_ 49.4) was obscured by overlapping with the solvent signal in the ^13^C NMR spectrum (Figure S2, Supporting Information File 1); however, its presence was confirmed by HSQC and HMBC correlations, as described below (Figures S2, S4, and S5, Supporting Information File 1).

**Table 1:** NMR spectroscopic data for rhodopyran (**1**) in CD_3_OD.

position	δ_C_^a^, type		δ_H_, mult (*J* in Hz)^b^	HMBC^b,c^	NOE

2	75.6,	C			
3	142.9,	CH	6.72, s	2, 4, 4a, 8, 11	8, 13
4	130.2,	C			
4a	47.0,	CH	2.36, dd (8.6, 3.2)	3, 4, 11, 14	7, 7a, 14
5	40.4,	CH	1.97, m		4a, 6α, 14
6	35.3,	CH_2_	2.53, ddd (13.0, 9.1, 9.1): α	4a, 5, 7, 7a, 12, 14	5, 6β
			1.44, ddd (13.0, 9.6, 6.2): β	4a, 5, 7, 7a, 12, 14	6α, 7, 14
7	49.4,	CH	3.16, ddd (9.6, 9.1, 4.6)	6, 7a, 12	4a, 6β, 7a, 14 (weak)
7a	77.2,	CH	4.36, dd (4.6, 3.2)	2, 4 (weak), 4a, 5, 6	4a, 7, 13
8	135.5,	CH	5.48, dd (15.4, 1.7)	2, 3, 10	3, 9, 10, 13
9	125.0,	CH	5.73, dq (15.4, 6.6)	2, 3, 10	3, 8, 10, 13
10	17.9,	CH_3_	1.67, dd (6.6, 1.7)	8, 9	8, 9
11	170.6,	C			
12	176.1,	C			
13	24.3,	CH_3_	1.27, s	2, 3, 8	3, 7a, 8, 9
14	21.1,	CH_3_	1.11, d (6.9)	4a, 5, 6	4a, 5, 6β

^a^Recorded at 125 MHz (reference δ_C_ 49.0). ^b^Recorded at 500 MHz (reference δ_H_ 3.31). ^c^HMBC correlations are from proton(s) stated to the indicated carbon.

COSY and HSQC data defined two main partial structures: a 1-propenyl moiety (H-8/H-9/H_3_-10) with a large *trans* vicinal coupling (*J* = 15.4 Hz), consistent with an *E* configuration across C-8/C-9, and a methyl-branched aliphatic spin system, H-4a/H-5(H3-14)/H_2_-6/H-7/H-7a, including the H-5/H_2_-6 and H-5/H_3_-14 COSY correlations (Figure 1). Key HMBC correlations established the connectivity of these fragments and located the two carboxyl groups. Correlations from H-8 and H-9 to C-2 and C-3, together with the correlation from H_3_-13 to C-2/C-3/C-8, supported the connectivity of the 1-propenyl substituent and Me-13 at C-2. A carboxyl group at C-4 was assigned by correlations from H-3 and H-4a to the carbonyl carbon C-11, and by correlations from H-3 and H-4a to C-4. Likewise, correlations from H-6α/H-6β and H-7 to C-12 positioned another carboxyl group at C-7. The linkage from C-5 to C-6 and the placement of Me-14 at C-5 were corroborated by correlations from H-6α/H-6β to C-5 and from H_3_-14 to C-6. In addition, reciprocal correlations between H-3 and C-4a and between H-4a and C-3, as well as correlations from H-7a to C-2 and C-4a, supported the fusion of the cyclopentane and pyran rings. A weak but observed HMBC correlation from H-7a to C-4 was also detected and is shown as a dashed arrow in Figure 1. These data completed the planar structure of **1**.

**Figure 2:**
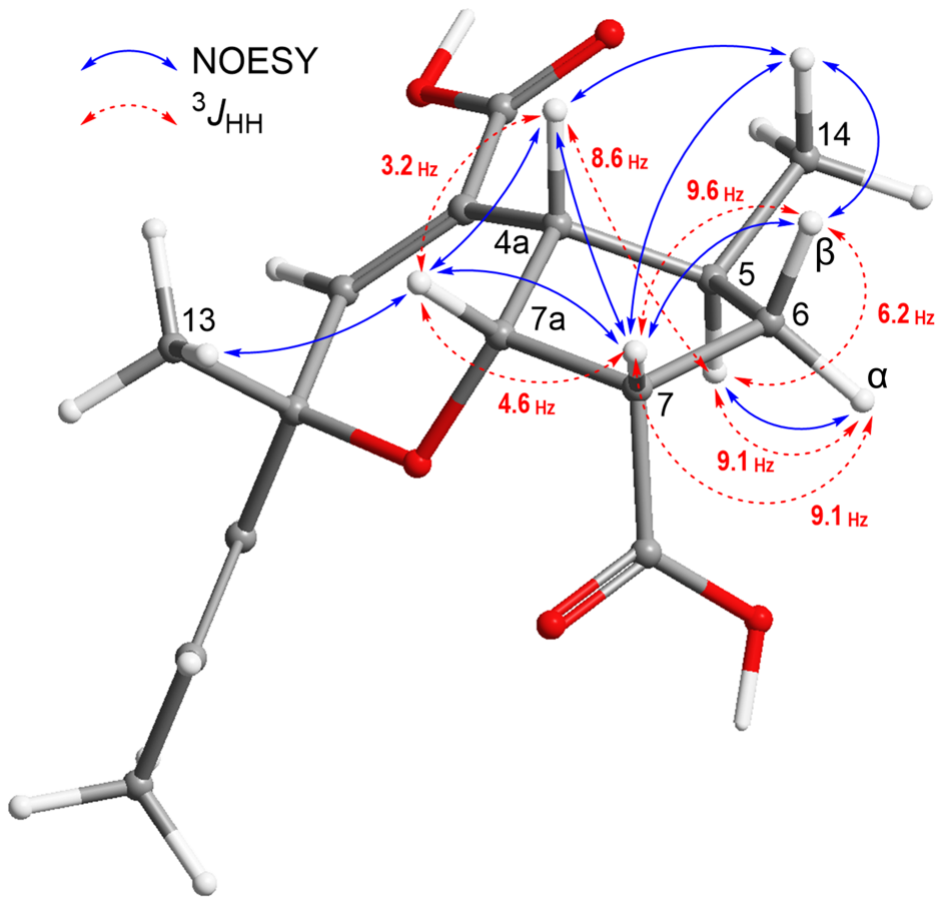
Selected vicinal ^3^*J*_HH_ values and key NOESY correlations used to assign the relative configuration of rhodopyran (**1**).

The relative configuration of **1** was determined by analysis of vicinal coupling constants around the bicyclic junction (H-4a, H-5, H-7a, H-7) and NOESY correlations (Figure 2). The large ^3^*J*_H-4a,H-5_ = 8.6 Hz supports an antiperiplanar alignment, whereas the small ^3^*J*_H-4a,H-7a_ = 3.2 Hz is consistent with a gauche relationship (ca. 60° dihedral angle). In addition, H-7a showed a moderate coupling to H-7 (*J* = 4.6 Hz), consistent with a gauche relationship. At C-6, H-6α showed two relatively large couplings to H-5 and H-7 (*J*_5,6α_ = 9.1 Hz and *J*_6α,7_ = 9.1 Hz), whereas H-6β exhibited a moderate coupling to H-5 (*J* = 6.2 Hz) and a large coupling to H-7 (*J* = 9.6 Hz). The pattern of vicinal couplings observed for the C-6 methylene protons is consistent with precedents for cyclopentane-containing natural products and conformationally restricted cyclopentane-based systems, in which diastereotopic methylene protons have been reported to exhibit multiple relatively large vicinal couplings, commonly attributed to ring puckering and conformational restriction [13–17]. In the NOESY spectrum, correlations among H-4a/H-7/H-7a and between H-7a and Me-13 indicated that these protons are located on the same face of the fused bicyclic framework. Diagnostic NOESY correlations further differentiated the C-6 protons: H-6β correlated with H-7, while H-6α correlated with H-5. An additional correlation between Me-14 and H-4a (and H-6β) supported the location of Me-14 on the same face. Thus, the relative configuration of rhodopyran (**1**) was established as shown in Figure 2. Attempts to determine the absolute configuration by ECD analysis and single-crystal X-ray diffraction were unsuccessful because the ECD spectrum was not sufficiently diagnostic and crystals suitable for diffraction could not be obtained; therefore, the absolute configuration remains unassigned.

To the best of our knowledge, more than 400 natural products containing a cyclopenta[*c*]pyran skeleton, predominantly iridoids, have been reported [16], whereas natural products featuring a cyclopenta[*b*]pyran skeleton are exceedingly rare [18–23]. Reported microbial natural products containing a cyclopenta[*b*]pyran motif include diospentenols A and B from *Streptomyces* sp. KMA-001, which possess a 7,7a-dihydro-2*H*-cyclopenta[*b*]pyran-6-one core bearing a hydroxy group and an allyl side chain [19]. In contrast, rhodopyran (**1**) is a carboxylated hexahydrocyclopenta[*b*]pyran bearing two carboxyl groups and a 1-propenyl substituent, and therefore differs markedly from the diospentenols in carbon number, oxidation state, and substitution pattern. These differences suggest that the diospentenols should be regarded as structural comparators rather than biosynthetic analogues of **1**. Moreover, the few reported examples differ substantially from compound **1** in that they are more highly oxidized (e.g., ketone-containing derivatives) and possess distinct architectures such as spirocyclic motifs or three-membered rings. Collectively, these observations suggest that the natural product chemical space of this structural class remains largely unexplored, and underscore that pursuing genome mining in parallel with cultivation-based screening of underexplored microbial taxa can directly contribute to expanding structural diversity.

**Scheme 1:**
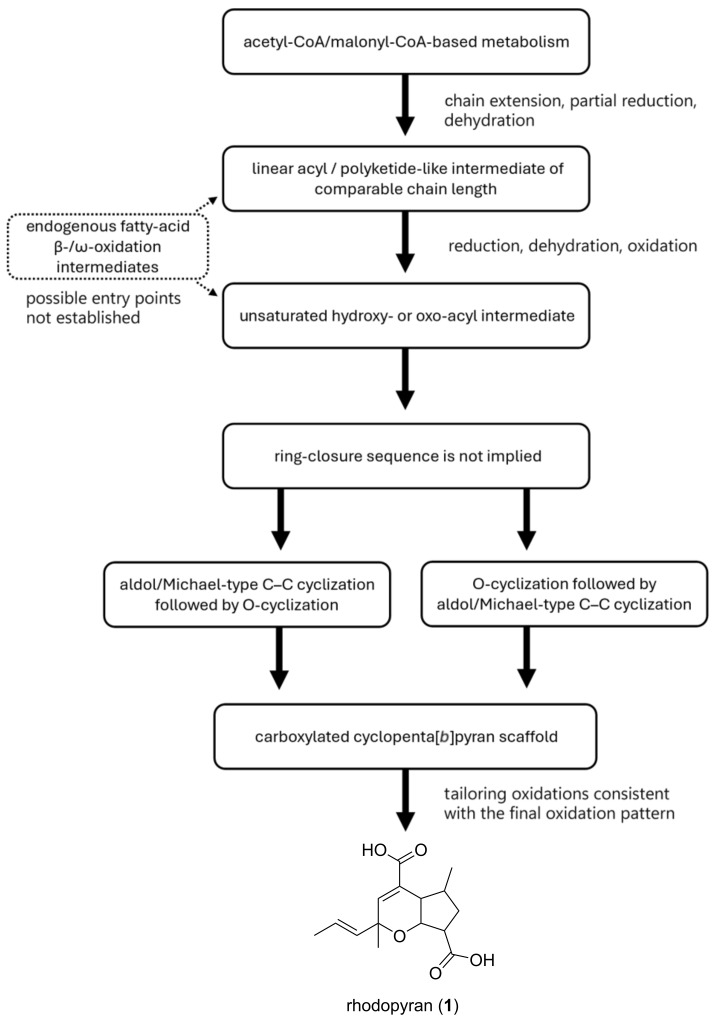
Tentative biosynthetic scenarios for rhodopyran (**1**). Intermediates are depicted as generic structural classes; regiochemistry, stereochemistry, carbon-by-carbon mapping, and the order of ring closure are not implied. The carbon framework of **1** could plausibly arise from a linear acyl- or polyketide-like intermediate derived from acetyl-CoA/malonyl-CoA-based metabolism, followed by partial reduction, dehydration, intramolecular aldol- or Michael-type C–C cyclization, intramolecular O-cyclization, and tailoring oxidations. Considering the oxidative and catabolic capacity of *Rhodococcus*, possible entry from endogenous fatty-acid β-/ω-oxidation intermediates cannot be excluded. The responsible enzymes and detailed pathway remain to be established.

The biosynthetic origin of rhodopyran (**1**) remains to be established (Scheme 1). From a structural viewpoint, the carbon framework of **1** could plausibly arise from a linear acyl- or polyketide-like intermediate derived from acetyl-CoA/malonyl-CoA-based metabolism. Partial reduction, dehydration, and oxidation of such an intermediate could generate an unsaturated hydroxy- or oxo-acyl precursor suitable for intramolecular ring formation. The cyclopentane ring may be formed through an intramolecular aldol- or Michael-type C–C cyclization, whereas closure of the pyran ring could proceed by intramolecular oxa-Michael addition or an epoxide-opening-type O-cyclization. The order of these two ring-forming events cannot be determined from the present data. An alternative catabolic contribution should also be considered. *Rhodococcus* is known for broad oxidative and biodegradative capabilities, and endogenous fatty-acid β-oxidation or ω-oxidation intermediates could, in principle, enter a partially dedicated biosynthetic route. Such a scenario is not mutually exclusive with a polyketide- or fatty-acyl-derived origin, because both routes could converge at the level of linear β-oxo/β-hydroxy acyl intermediates. The absence of **1** from the uninoculated A-3M medium control indicates that rhodopyran is not a pre-existing medium component or a medium-derived extraction artifact. However, the responsible enzymes, the order of ring closure, and the carbon assembly pattern remain unknown. Genome sequencing, biosynthetic gene cluster analysis, isotope-labeling experiments, and time-course metabolite profiling will be required to clarify the origin of **1**.

## Conclusion

In summary, rhodopyran (**1**) is a structurally distinctive carboxylated hexahydrocyclopenta[*b*]pyran isolated from *Rhodococcus* sp. RD066637. Its planar structure and relative configuration were established by HRMS and detailed NMR analysis, whereas the absolute configuration remains unassigned. This bicyclic scaffold expands the structural diversity of natural products and underscores the potential of *Rhodococcus* as a source of uncommon chemical architectures.

## Experimental

### General experimental procedures

Optical rotations were measured using a DIP-3000 polarimeter (JASCO, Tokyo, Japan). UV spectra were recorded on a UV-1900 spectrophotometer (Shimadzu, Kyoto, Japan). IR spectra were measured by a spectrum 100 spectrometer (PerkinElmer, MA, USA). NMR spectra were obtained on an AVANCE NEO 500 spectrometer (Bruker, MA, USA) in CD_3_OD and referenced to the residual solvent signals (δ_H_ 3.31, δ_C_ 49.0). HRESIQTOFMS spectra were recorded on a compact QTOF mass spectrometer (Bruker). HPLC separations were performed using a COSMOSIL 5C_18_-AR-II Packed Column (10 × 250 mm, Nacalai Tesque, Inc., Kyoto, Japan).

### Microorganism

The strain RD066637 was obtained from Biological Resource Center, National Institute of Technology and Evaluation, Japan. The RD strain refers to a collection of strains from various domestic and international sources provided by NBRC for screening purposes. Detailed strain information is available under DDBJ accession number LC688269. This study did not involve access to foreign genetic resources subject to the Nagoya Protocol. The 16S rRNA gene sequence of the strain showed 99.3% homology with the closely related *Rhodococcus cerastii* C5^T^ (FR714842), leading to its identification as belonging to the genus *Rhodococcus* (1466 nucleotides; DDBJ accession number LC688269).

### Fermentation

Strain RD066637 growing on an ISP 2 agar medium consisting of 0.4% yeast extract (Kyokuto Pharmaceutical Industrial, Tokyo, Japan), 1.0% malt extract (Becton Dickinson, NJ, USA), 0.4% glucose, 1.5% agar (pH 7.2) was inoculated into 500 mL K-1 flasks (custom-designed cylindrical flasks, cylindrical section: 75 mm inner diameter × 80 mm height; opening: 25 mm inner diameter) each containing 100 mL of the ISP 2 seed medium. The flasks were placed on a rotary shaker (200 rpm) at 30 °C for 2 days. Then, the seed culture (3 mL) was transferred into 500 mL K-1 flasks, each containing 100 mL of the A-3M production medium consisting of 2.0% soluble starch, 2.0% glycerol, 0.5% glucose, 1.5% Pharmamedia (Archer-Daniels-Midland Company, TX, USA), 0.3% yeast extract, and 1.0% Diaion HP-20 resin (for improvement of specialized metabolites productivity, Mitsubishi Chemical, Tokyo, Japan) in distilled water. The pH of the medium was adjusted to 7.0 before sterilization. All the media were sterilized by autoclaving at 121 °C for 20 min. The inoculated 20 flasks were placed on a rotary shaker (200 rpm) at 30 °C for 5 days.

### Extraction and isolation

In a manner similar to our previous report [24], extraction and isolation were carried out as follows. After incubation, 100 mL of 1-BuOH was added to each flask, and the flasks were allowed to shake for 1 h. The mixture was centrifuged at 6,000 rpm for 10 min, and the organic layer was separated from the aqueous layer containing the mycelium. The BuOH layer was evaporated to give 12.6 g of extract from 2 L of culture. The extract was fractionated using silica gel column chromatography with a step gradient of CHCl_3_–MeOH (1:0, 20:1, 10:1, 4:1, 2:1, 1:1, and 0:1 v/v). The fraction 4:1 containing **1** was concentrated to give 1.2 g of yellowish black solid, which was subjected to preparative HPLC using isocratic conditions of 30% aqueous MeCN containing 0.1% HCO_2_H at 4 mL/min, yielding rhodopyran (**1**, 22.7 mg) with a retention time of 11.2 min.

Rhodopyran (**1**): colorless amorphous powder; [α]_D_^25^ −69 (*c* 0.01, MeOH); UV (MeOH) λ_max_ (log ε) 217 nm (3.93); IR (ATR) ν_max_ 2965, 2928, 1688, 1640, 1421 cm^−1^; ^1^H and ^13^C NMR data, Table 1 and Supporting Information File 1; HRESIQTOFMS (negative ion mode) *m*/*z* 279.1237 [M − H]^−^ (calcd for C_15_H_19_O_5_^−^, 279.1238; Δ −0.4 ppm).

## Supporting Information

File 1 NMR, HRMS, IR spectra, and HPLC chromatogram for compound **1**.

## Data Availability

All data supporting the findings of this study are available in the article and its Supporting Information. The 16S rRNA gene sequence of strain RD066637 is available in DDBJ under accession number LC688269.
