# Supplementary material for: Rhodopyran, a carboxylated hexahydrocyclopenta[b]pyran from Rhodococcus
Source: Beilstein J Org Chem. 2026 Jul 28;22:1107–13. doi: 10.3762/bjoc.22.89 (PMC13430524; doi:10.3762/bjoc.22.89)

BEILSTEIN JOURNAL OF ORGANIC CHEMISTRY

Supporting Information

for

Rhodopyran, a carboxylated hexahydrocyclopenta[*b*]pyran from *Rhodococcus*

Enjuro Harunari, Sara Fushimi and Yasuhiro Igarashi

Beilstein J. Org. Chem. **2026,** *22,* 1107–1113. doi:10.3762/bjoc.22.89

NMR, HRMS, IR spectra, and HPLC chromatogram for compound 1

License and Terms: This is a supporting information file under the terms of the Creative Commons Attribution License (https://creativecommons.org/licenses/by/4.0). Please note that the reuse, redistribution and reproduction in particular requires that the author(s) and source are credited and that individual graphics may be subject to special legal provisions.
The license is subject to the *Beilstein Journal of Organic Chemistry* terms and conditions: (https://www.beilstein-journals.org/bjoc/terms)

Table of Contents

Figure S1. ^{1}H NMR spectrum of rhodopyran (**1**) (500 MHz, CD_3OD)
Figure S2. ^{13}C NMR spectrum of **1** (125 MHz, CD_3OD)
Figure S3. ^{1}H-^{1}H COSY spectrum of **1** (500 MHz, CD_3OD)
Figure S4. HSQC spectrum of **1** (500 MHz, CD_3OD)
Figure S5. HMBC spectrum of **1** (500 MHz, CD_3OD)
Figure S6. NOESY spectrum of **1** (500 MHz, CD_3OD)
Figure S7. HRMS spectrum of **1**
Figure S8. IR spectrum of **1**
Figure S9. HPLC chromatograms of BuOH extracts from the RD066637 culture and the uninoculated A-3M medium control.

Figure S1. ^{1}H NMR spectrum of **1** (500 MHz, CD_3OD)

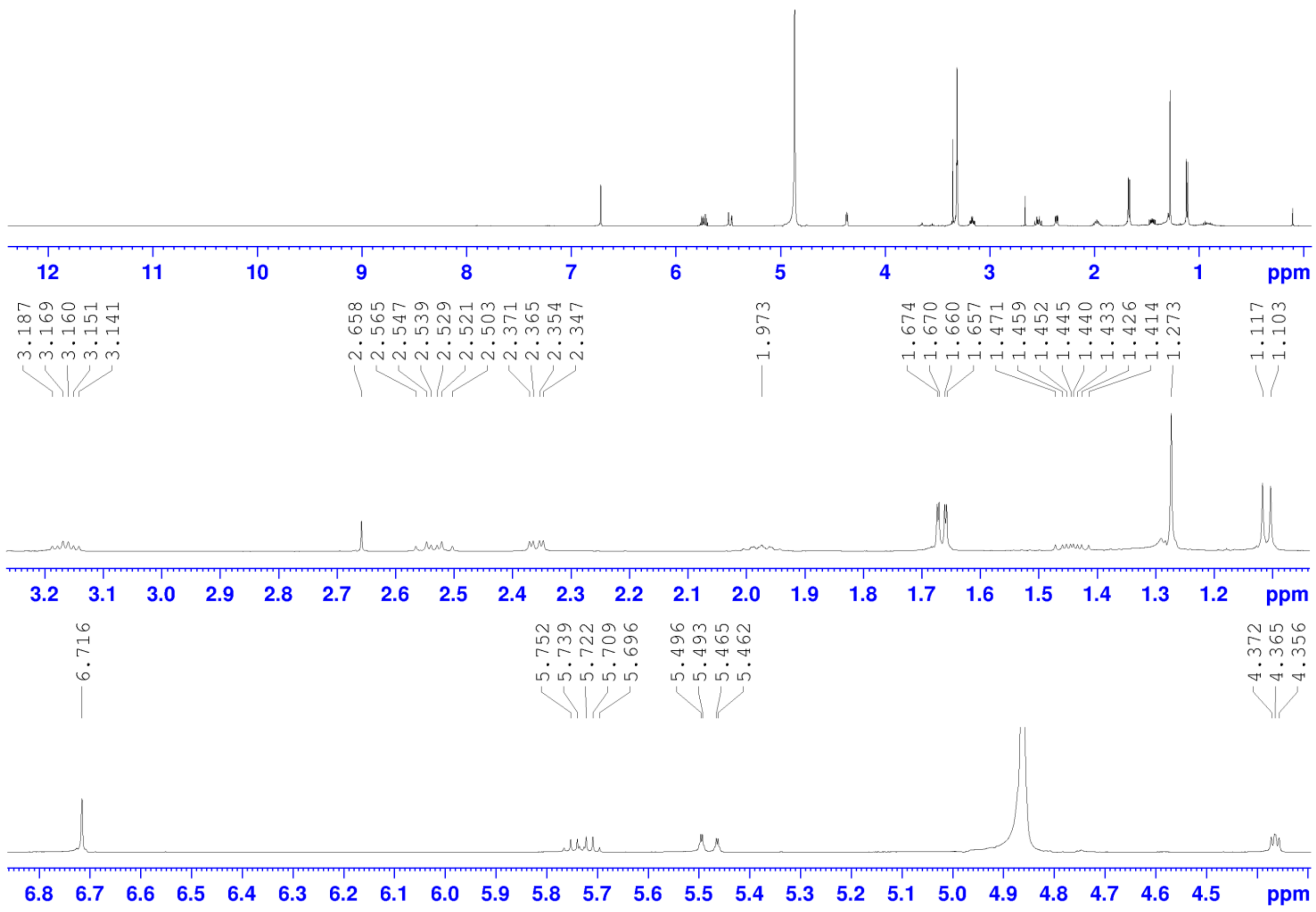

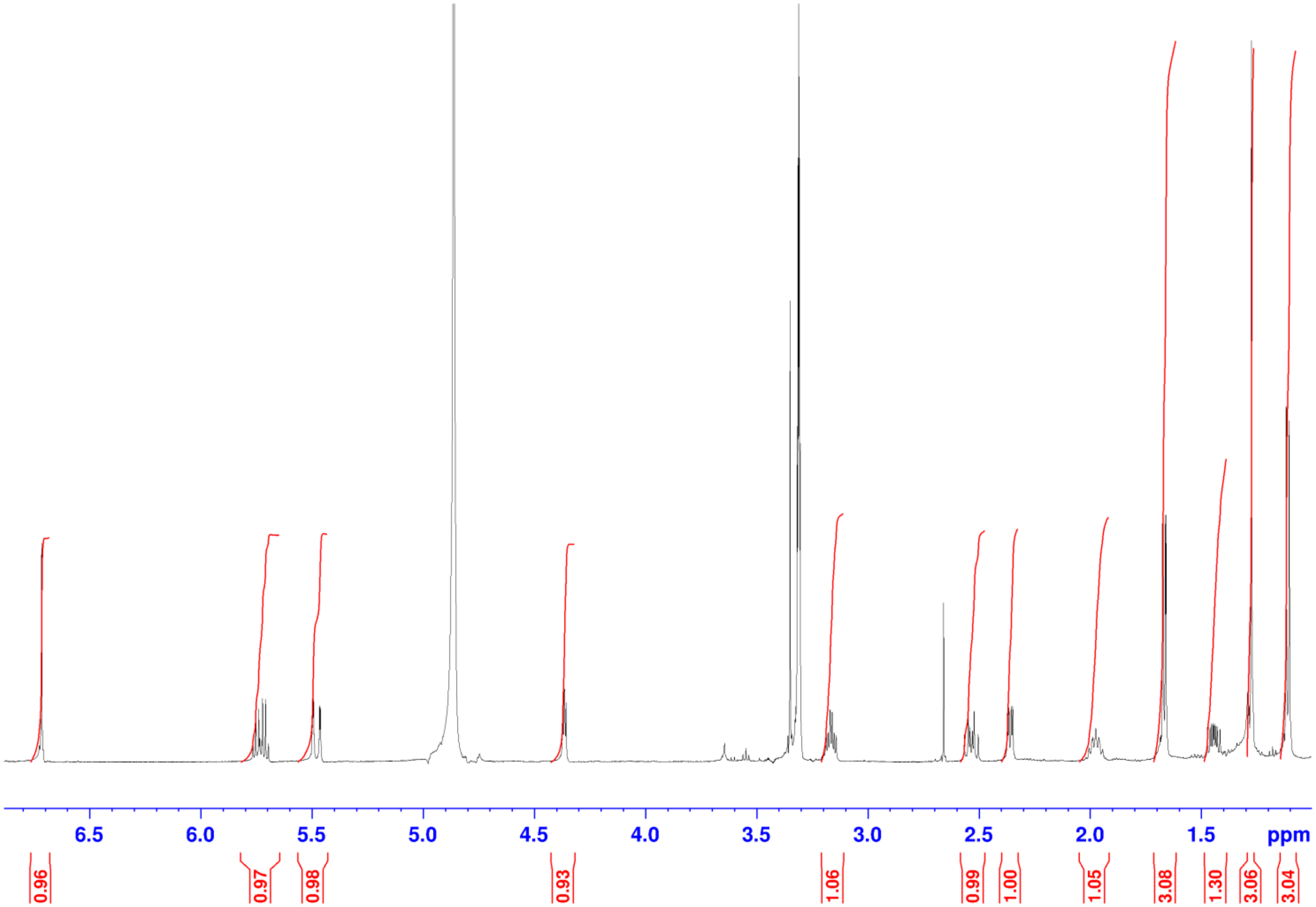

6.5
6.0
5.5
5.0
4.5
4.0
3.5
3.0
2.5
2.0
1.5
ppm
0.96
0.97
0.98
0.93
1.06
0.99
1.00
1.05
3.08
1.30
3.06
3.04

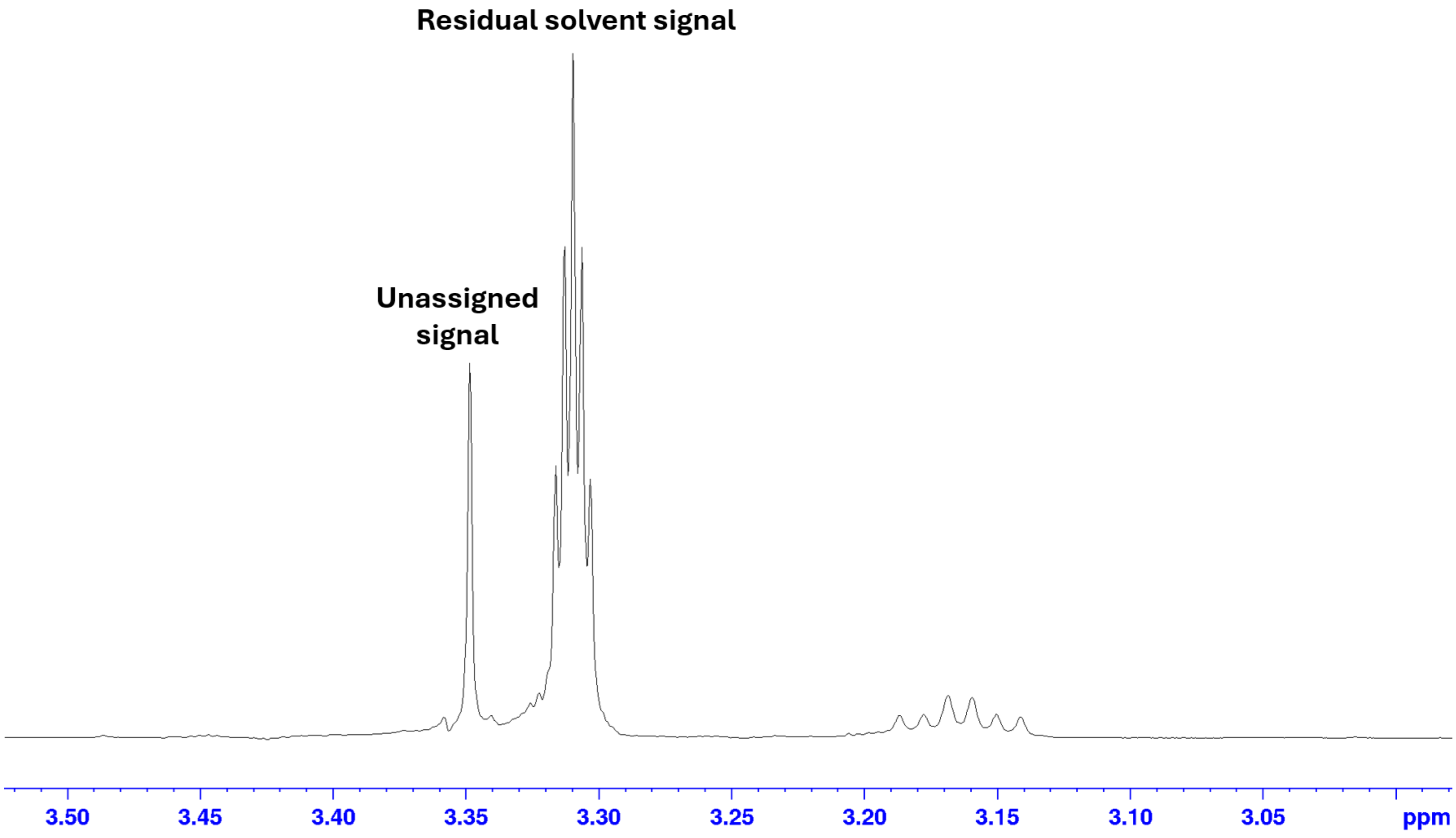

Residual solvent signal
Unassigned signal
3.50
3.45
3.40
3.35
3.30
3.25
3.20
3.15
3.10
3.05
ppm

Figure S2. ^{13}C NMR spectrum of **1** (125 MHz, CD_3OD)

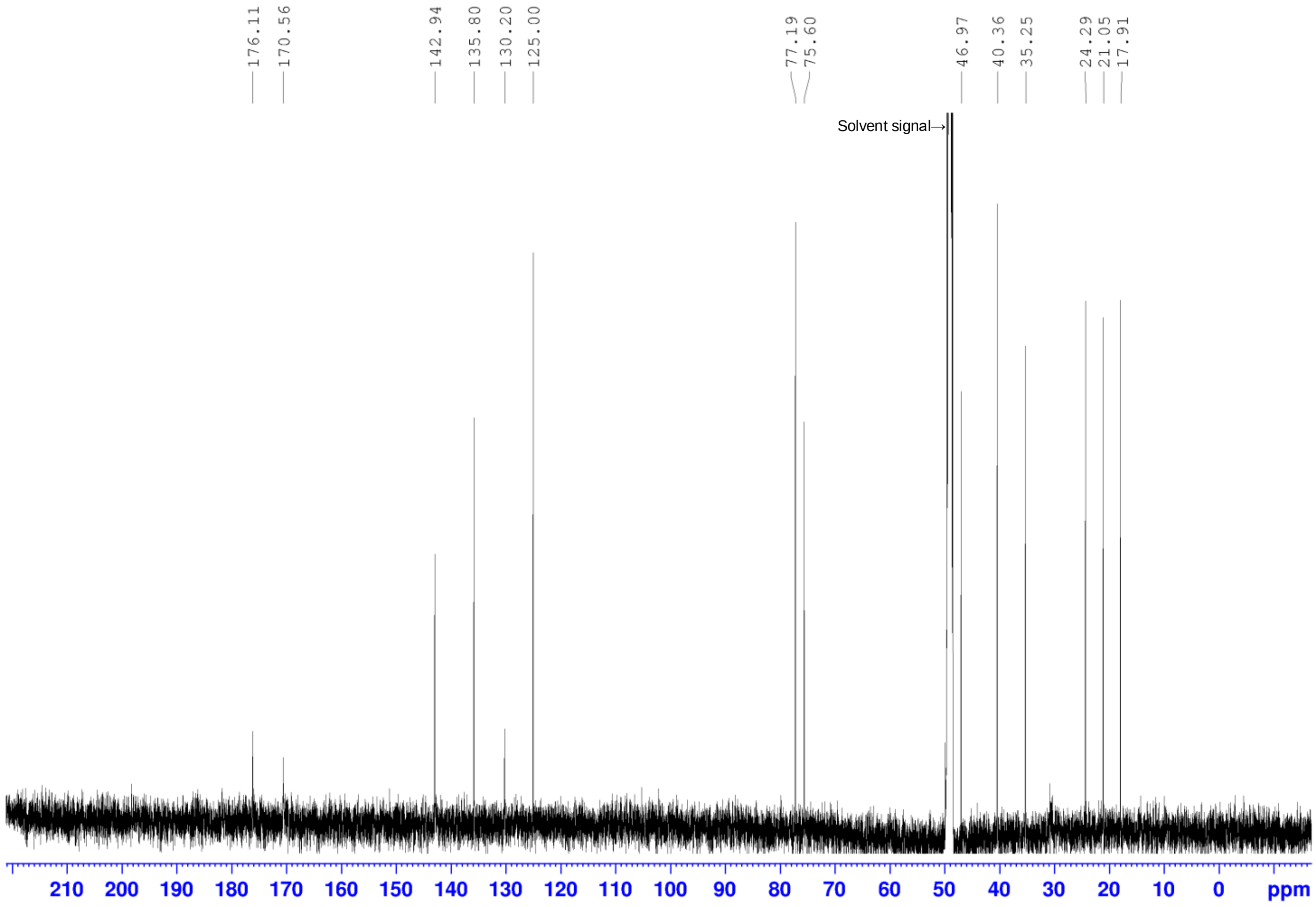

Figure S3. ^{1}H-^{1}H COSY spectrum of **1** (500 MHz, CD_3OD)

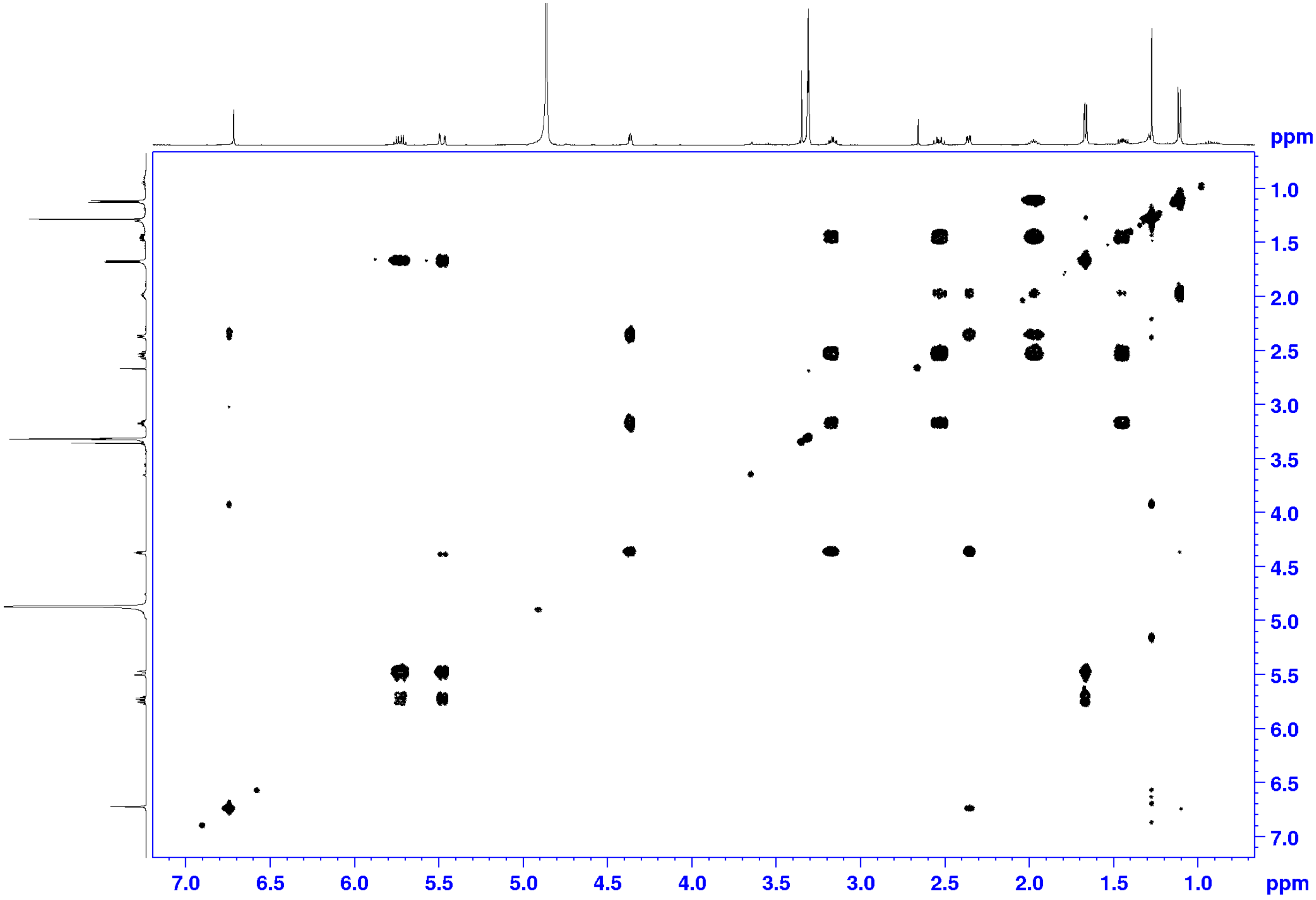

Figure S4. HSQC spectrum of **1** (500 MHz, CD_3OD)

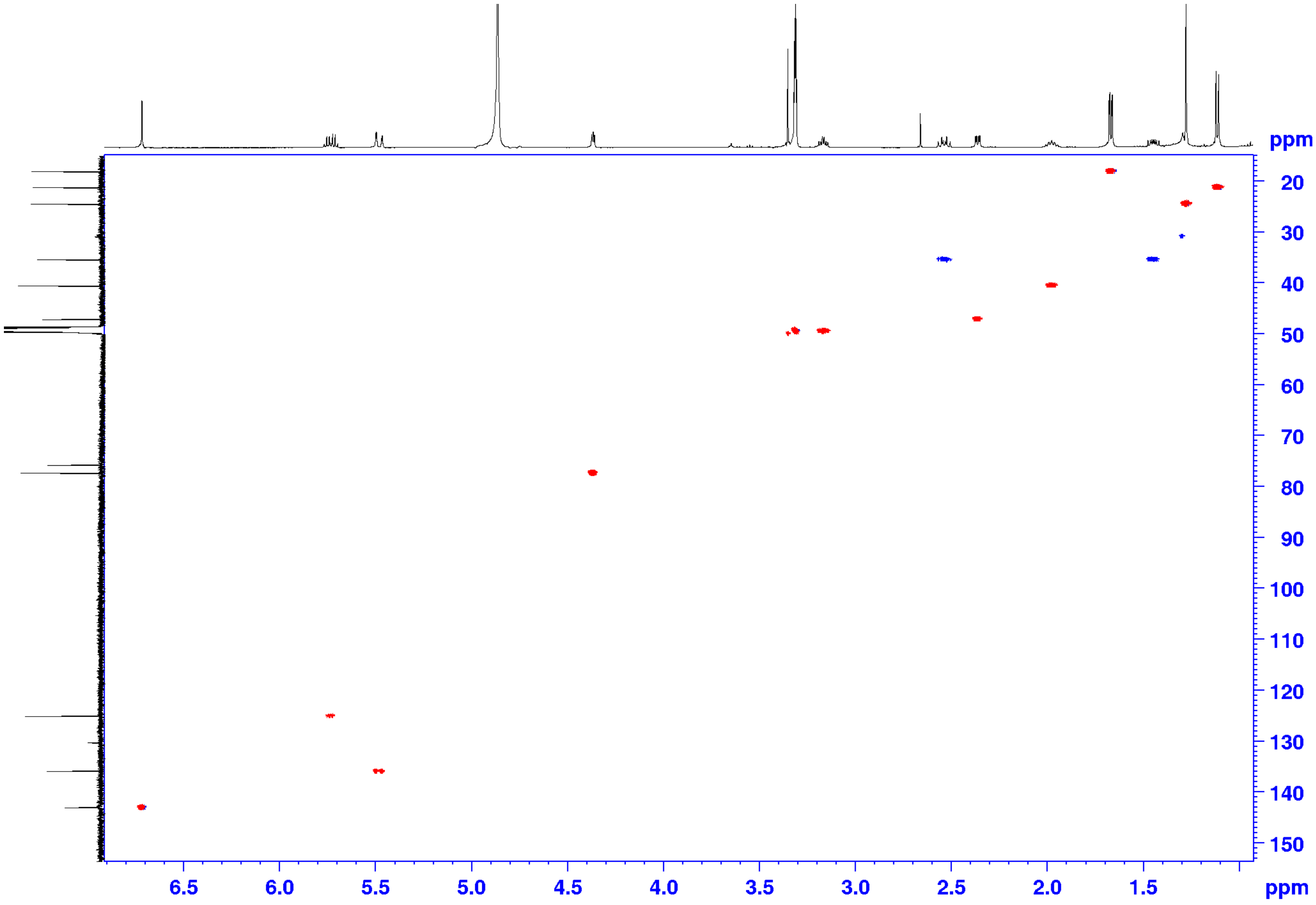

Figure S5. HMBC spectrum of **1** (500 MHz, CD_3OD)

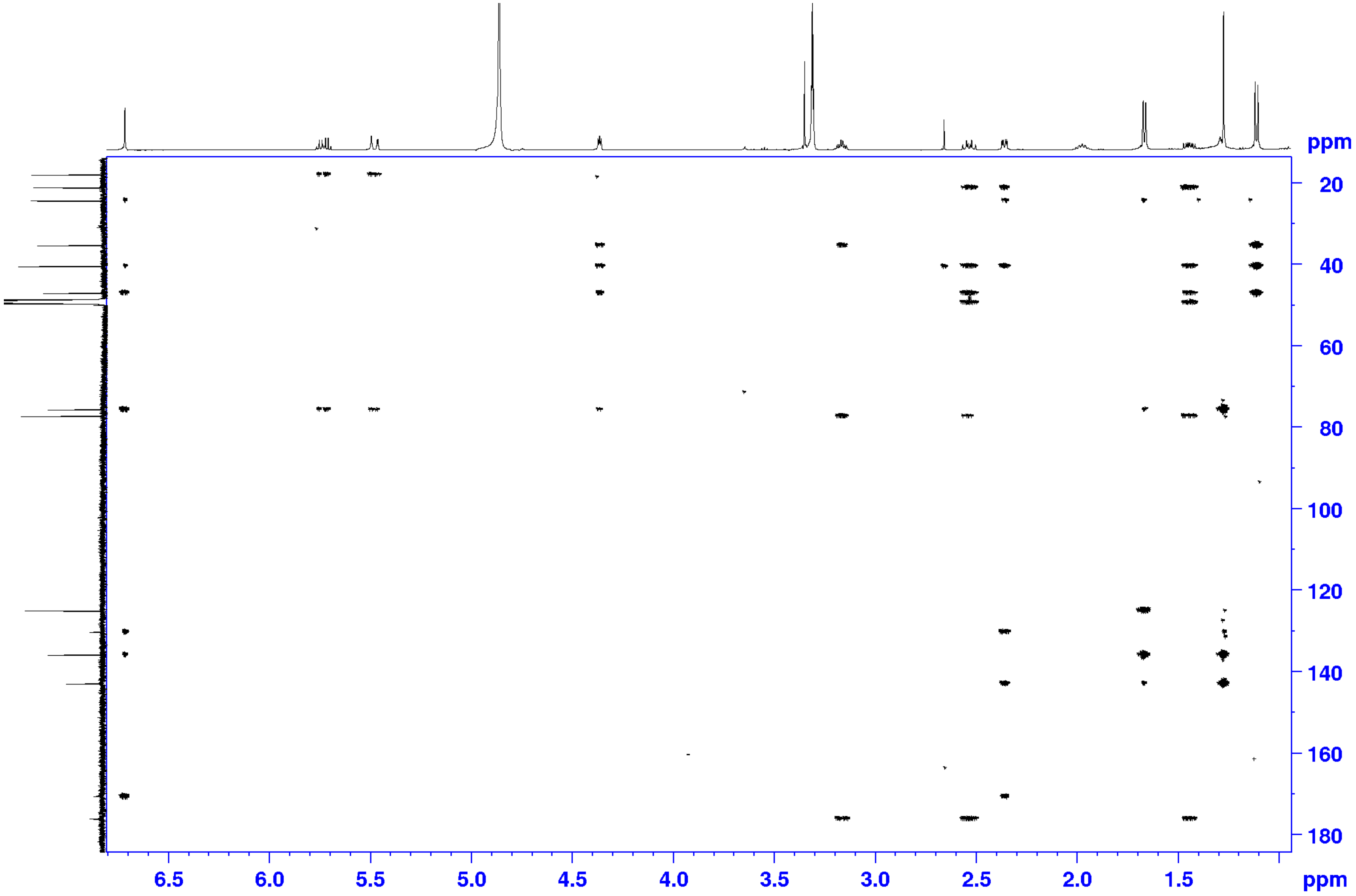

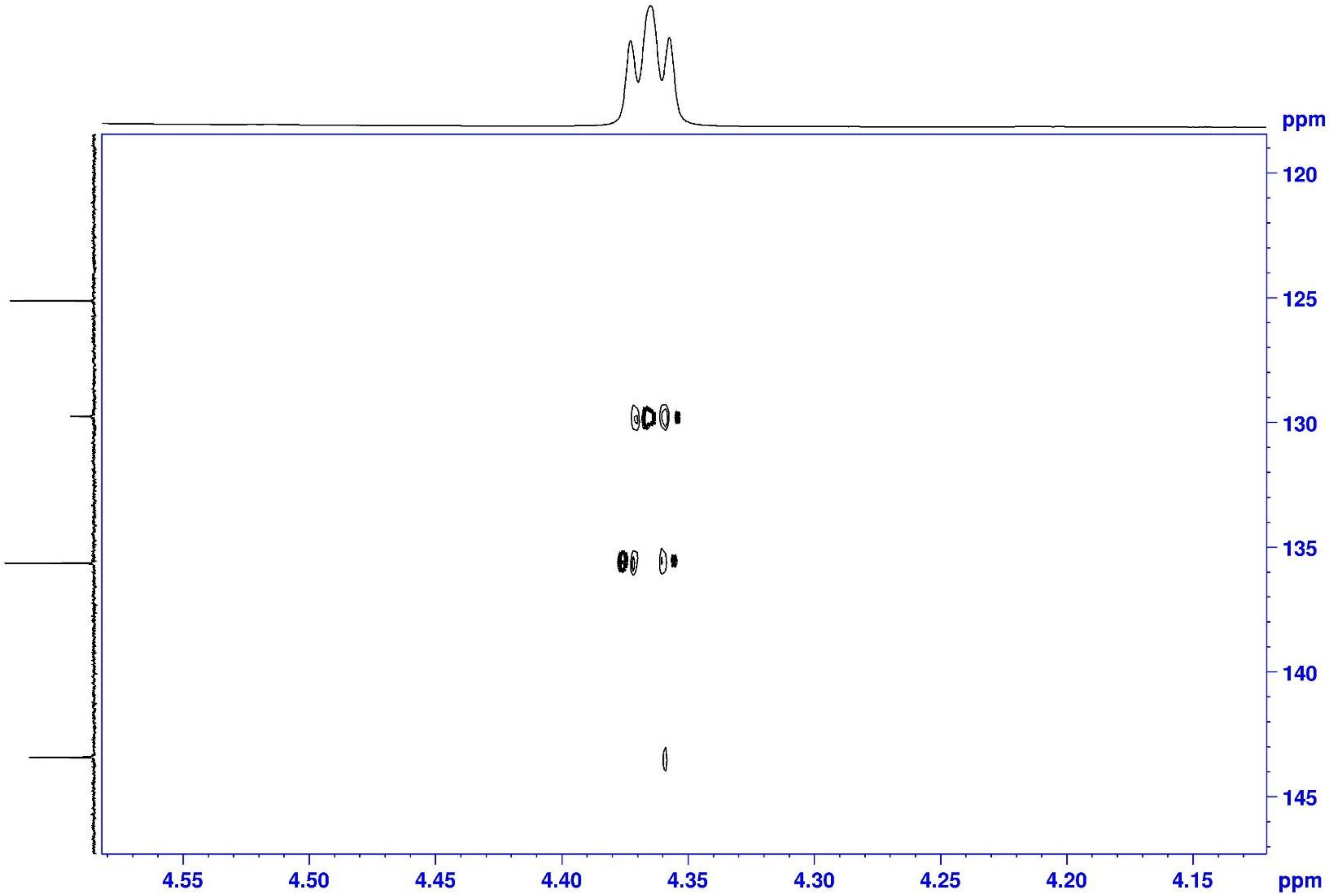
ppm
120
125
130
135
140
145
4.55
4.50
4.45
4.40
4.35
4.30
4.25
4.20
4.15
ppm

Figure S6. NOESY spectrum of **1** (500 MHz, CD_3OD)

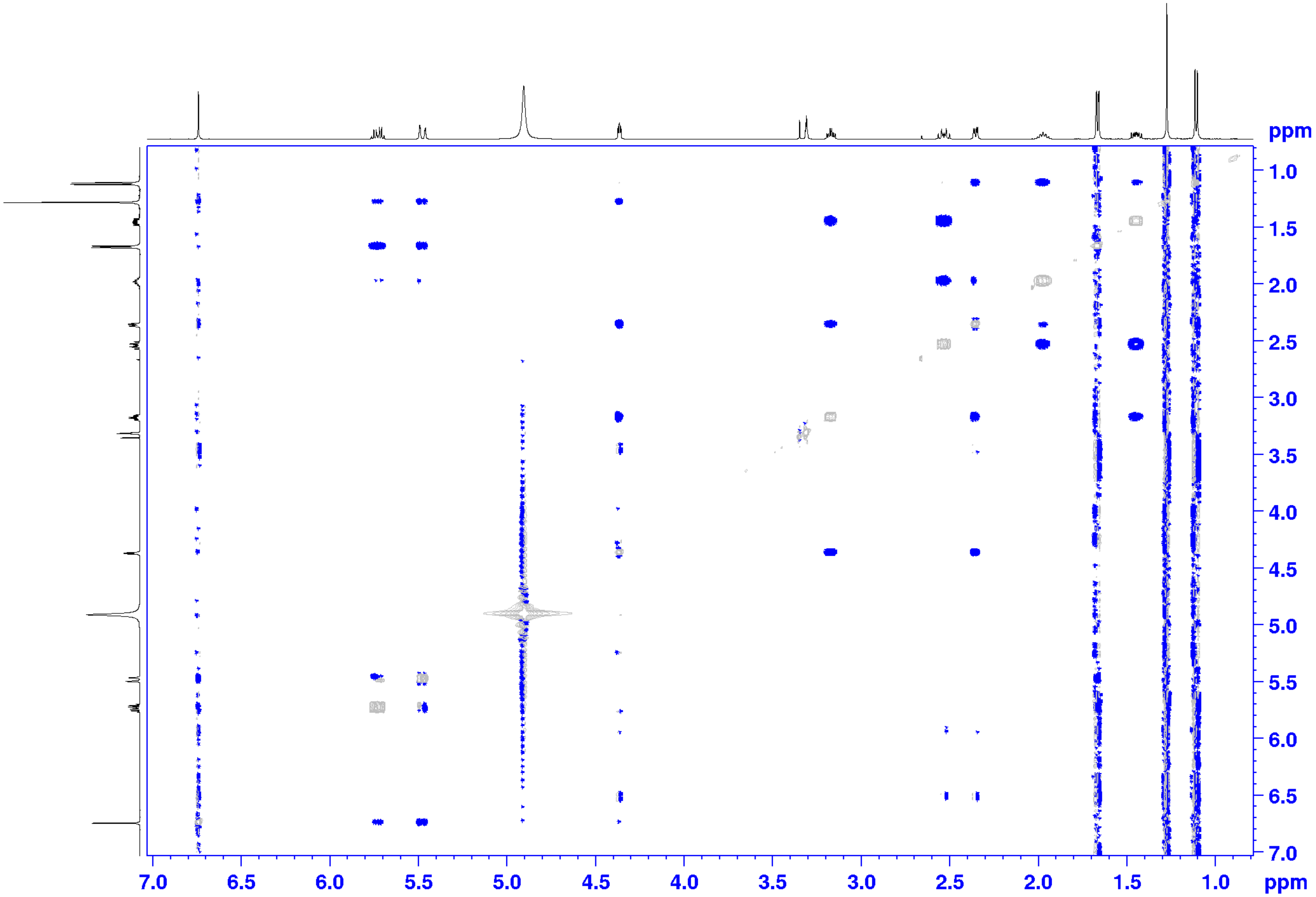

Figure S7. HRMS spectrum of **1**. The ion at *m*/*z* 279.1237 corresponds to [M–H]$^{-}$ of 1; other intense ions are attributable to background/impurity ions.

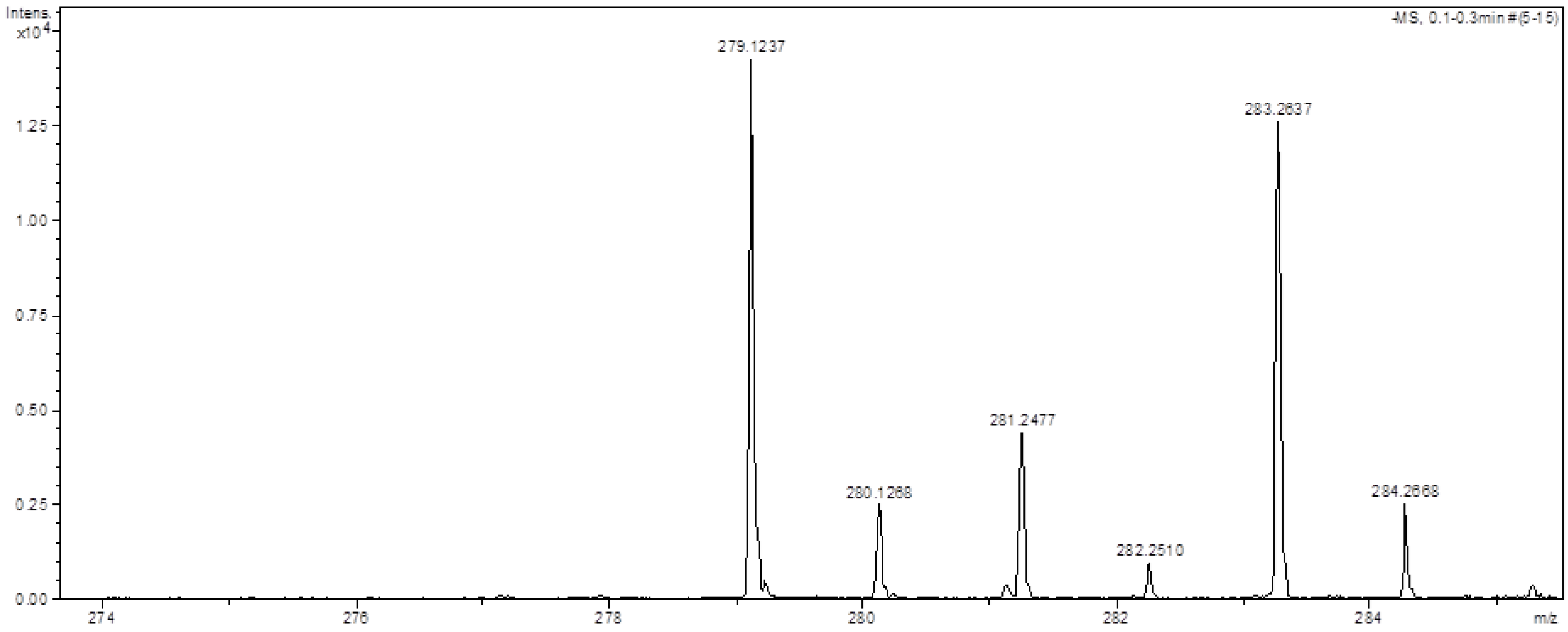

Figure S8. IR spectrum of **1**.

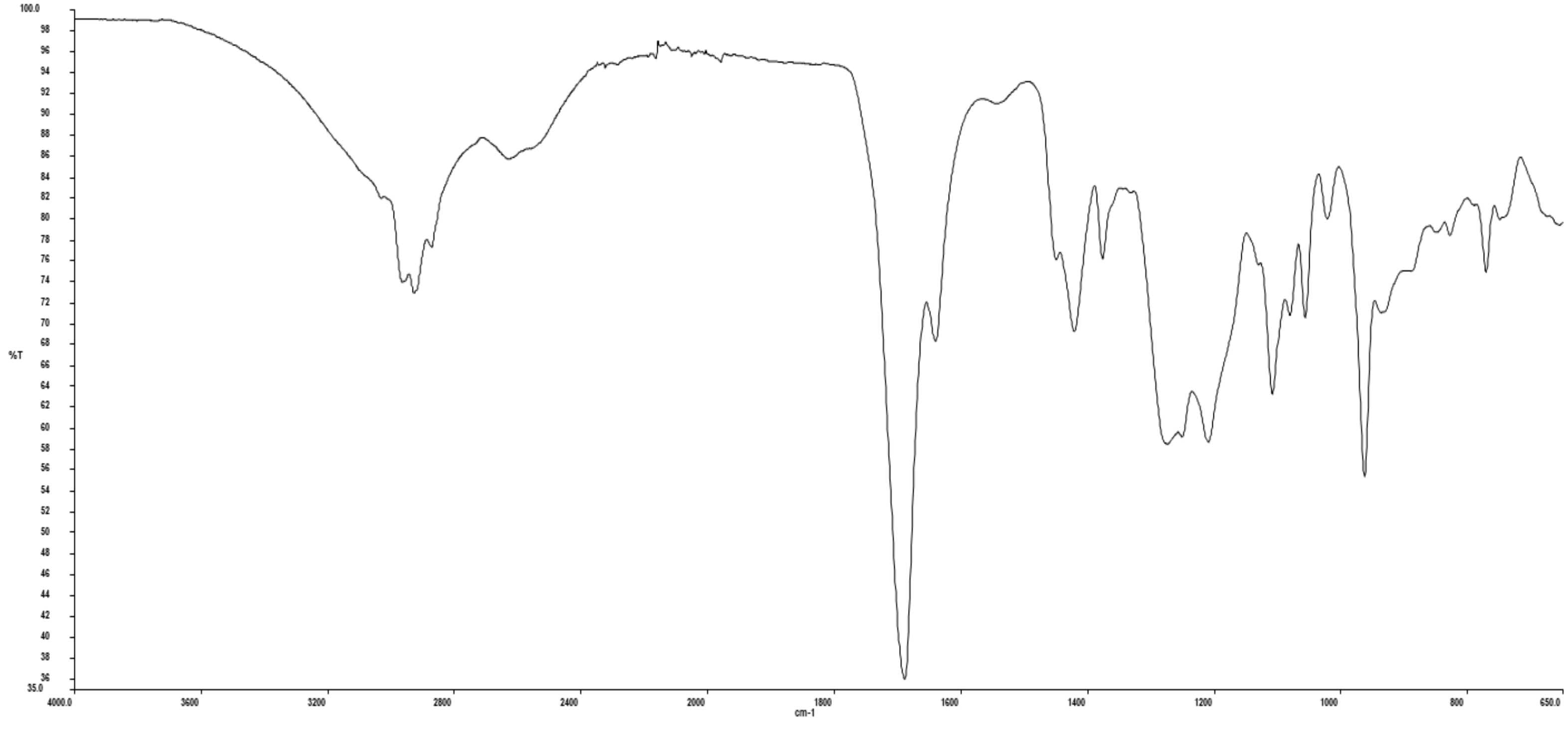

Figure S9. HPLC chromatograms of BuOH extracts from the RD066637 culture and the uninoculated A-3M medium control. (A) *Rhodococcus* sp. RD066637 cultured in A-3M medium for 5 days. (B) Uninoculated A-3M medium incubated for 5 days under identical conditions. Both samples were processed in the same manner, including 1-BuOH extraction. The red arrow indicates rhodopyran (**1**). No peak corresponding to **1** was detected in the medium control. The HPLC analysis was performed using a C18-AR II column (4.6 × 100 mm) with water and acetonitrile as the mobile phases at a flow rate of 1.2 mL/min. The gradient program was as follows: 15% MeCN from 0 to 3 min, a linear increase to 85% MeCN from 3 to 25 min, 85% MeCN from 25 to 28 min, and a return to 15% MeCN by 32 min. UV detection was performed at 230 nm.

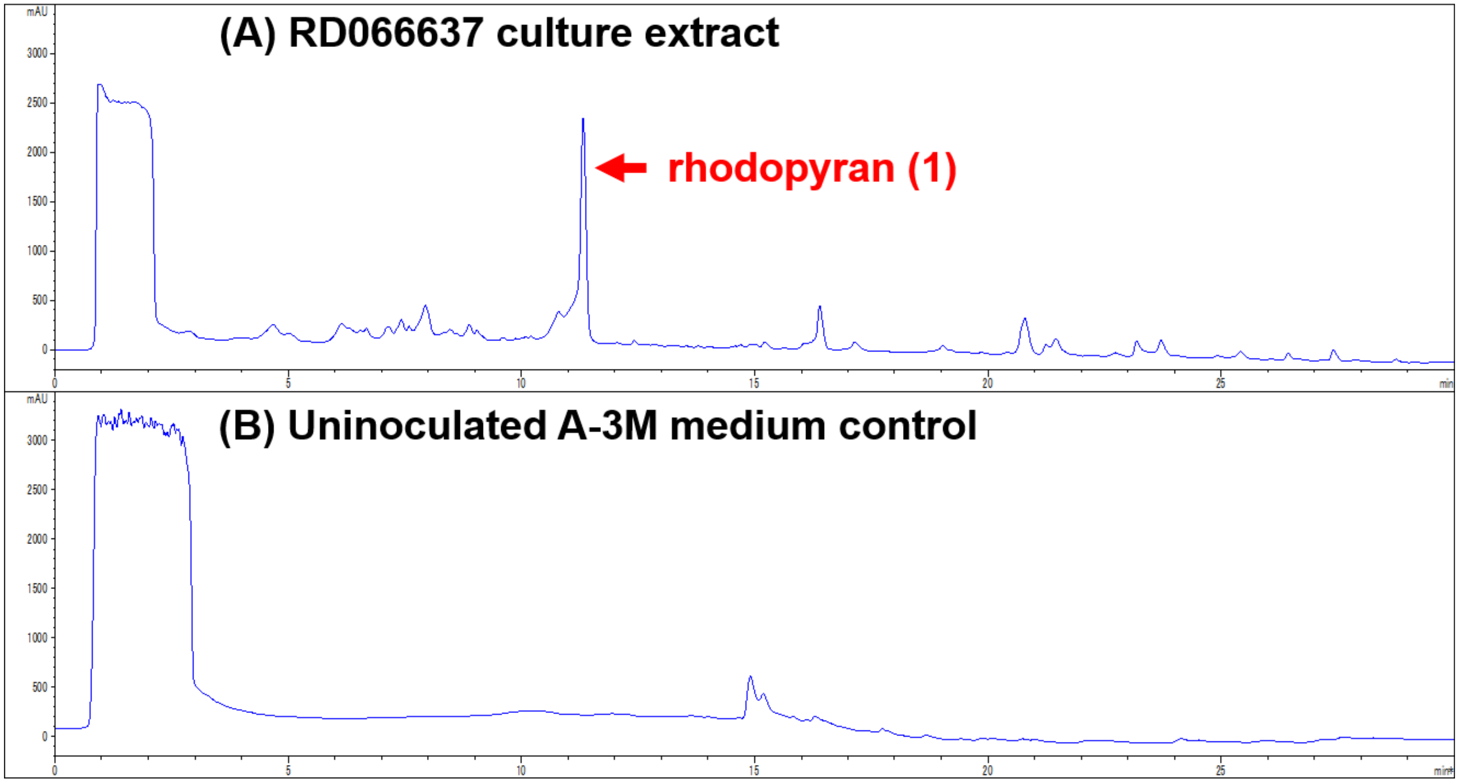

Supplement: File 1 — NMR, HRMS, IR spectra, and HPLC chromatogram for compound 1. [file Beilstein_J_Org_Chem-22-1107-s001.pdf]
